# A bioinspired enzyme-responsive hydrogel for spatiotemporal control of macrophage reprogramming in chronic wounds

**DOI:** 10.3389/fbioe.2026.1825302

**Published:** 2026-04-30

**Authors:** Tao Jing

**Affiliations:** School of Science, Xichang University, Xichang, China

**Keywords:** chronic wounds, diabetic wound healing, enzyme-responsive hydrogels, M1/M2 polarization, macrophage reprogramming, spatiotemporal control

## Abstract

**Background:**

Chronic wounds remain a significant challenge in healthcare due to persistent inflammation and disrupted macrophage polarization.

**Objective:**

In this study, we conducted a quantitative comparative analysis to assess how enzyme-responsive hydrogels influence macrophage behaviour and promote wound healing.

**Methods:**

A structured comparative dataset was constructed from 182 screened studies, with eight preclinical investigations included for quantitative analysis, focusing on hydrogels responsive to matrix metalloproteinases (MMP), reactive oxygen species (ROS), and cathepsins.

**Results:**

Wound closure rates ranged from 60% to 90% by day 14. ROS-responsive hydrogels accelerated early-stage healing, while MMP-responsive hydrogels provided more sustained effects. Hydrogels containing exosomes produced the best healing outcomes, while those containing nanozymes provided prolonged anti-inflammatory benefits. All hydrogel systems increased M2 macrophage marker expression and reduced pro-inflammatory cytokine levels. We identified key pathways, including redox regulation, extracellular matrix remodelling, and epigenetic mechanisms, that affect macrophage polarization.

**Conclusion:**

Preclinical results are promising, challenges such as the lack of standardized measurement protocols and limited clinical studies remain. This study offers a data-driven framework for understanding enzyme-responsive hydrogels and identifies priorities for future research.

## 1 Introduction

Chronic wounds are a growing global health problem, affecting over 40 million people and costing more than $25 billion each year. These wounds do not heal within 12 weeks and are often linked to conditions like diabetes, venous insufficiency, pressure injuries, and peripheral vascular disease. Diabetic foot ulcers are especially serious, with about one in four people with diabetes developing them at some point (Cao et al., 2023). Severe cases can lead to lower-extremity amputation in up to 20% of patients, which greatly increases illness, death rates, and lowers quality of life. As diabetes becomes more common and populations age, chronic wounds are also on the rise. This trend shows there is an urgent need for new treatments that address the root causes of poor healing.

### 1.1 Macrophage Dysfunction in chronic wounds

Chronic wounds often fail to heal because of ongoing inflammation and problems with the immune response. These issues prevent wounds from moving through the normal stages of repair: stopping bleeding, inflammation, new tissue growth, and remodeling. Macrophages play a key role in this process, and when they do not work properly, wound healing is affected. Normally, macrophages change their behavior as healing progresses (Zhang et al., 2023). At first, they take on a pro-inflammatory M1 form to remove pathogens and debris. Later, they switch to an anti-inflammatory M2 form that helps new blood vessels grow, supports tissue building, and aids in remodeling. This switch from M1 to M2 usually happens within the first week after injury and is crucial for ending inflammation and starting tissue growth.

In chronic wounds, especially diabetic ulcers, this important switch in macrophage behavior does not happen as it should. Macrophages stay in the pro-inflammatory M1 state, where they keep producing inflammatory cytokines like tumor necrosis factor-α, interleukin-1β, and interleukin-6, as well as reactive oxygen species and matrix metalloproteinases (Basheer et al., 2021). This ongoing M1 activity creates a harmful environment with too much oxidative stress, breakdown of the tissue matrix, poor blood vessel growth, and slow skin repair. On the other hand, M2 macrophages, which show markers like CD206 and arginase-1 and release anti-inflammatory cytokines such as interleukin-10 and transforming growth factor-β, are lacking in chronic wounds. Problems with the M1-to-M2 switch in diabetic wounds are linked to high blood sugar, buildup of advanced glycation end products, and constant activation of inflammatory pathways. Understanding that problems with macrophage behavior are a key feature of chronic wounds has led to new biomaterials that aim to guide macrophages toward healing (Xin et al., 2025).

**FIGURE 1:**
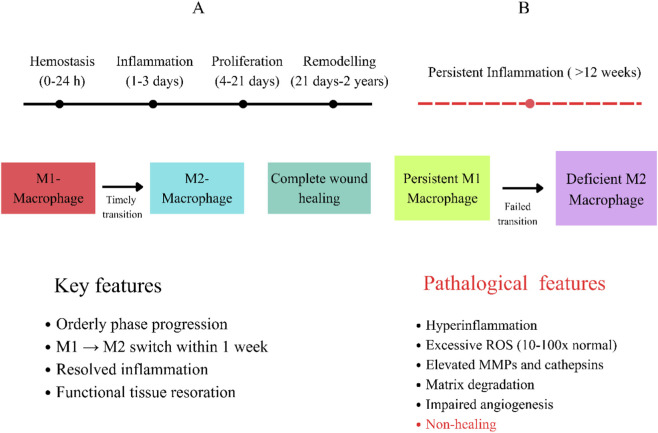
Chronic wound pathophysiology and macrophage dysfunction. **(A)** Normal wound healing. **(B)** Chronic wound (Impaired healing).

Figure 1 shows the difference between normal wound healing, where M1 macrophages change to M2 macrophages on time (Panel A), and chronic wounds, where M1 macrophages stay longer and there are fewer M2 macrophages (Panel B). The figure also points out important inflammatory markers and healing results.

### 1.2 Current therapeutic approaches and limitations

Current treatments for chronic wounds include a range of options, from traditional moist dressings and debridement to newer biomaterial-based therapies. Common methods use moisture-retentive dressings like hydrocolloids, alginates, and foams, as well as antimicrobial agents such as silver and iodine, negative-pressure wound therapy, and bioengineered skin substitutes. Still, these treatments often do not address the immune problems and ongoing inflammation seen in chronic wounds. While standard dressings help maintain moisture and act as a barrier, they do not actively influence the immune response or control when and where treatments are delivered. Growth factor therapies, such as recombinant platelet-derived growth factor, have only shown limited success because they break down quickly in the wound environment and cannot change the inflammatory conditions (Zhao et al., 2025).

Newer biomaterial approaches, especially hydrogel-based dressings, show promise because they are compatible with the body, can be adjusted for different needs, deliver drugs over time, and imitate parts of the natural tissue structure. Recent developments include hydrogels that contain antimicrobial agents, ROS scavengers, growth factors, stem cells, and extracellular vesicles (Li et al., 2024). However, there are still important challenges. These include not being able to control when and where treatments are released to match the changing wound environment, difficulty balancing infection control with encouraging healing, unwanted effects from constant drug release, poor response to the wound’s specific conditions, and limited ability to change macrophage behavior (Firlar et al., 2022). These issues highlight the need for smarter biomaterials that can detect wound-specific problems and deliver immune therapies as needed to support proper healing.

### 1.3 Enzyme-responsive hydrogels

Enzyme-responsive hydrogels are a promising approach for treating chronic wounds. They can sense high levels of certain enzymes or chemical signals found in unhealthy wounds and respond by changing their structure, breaking down, or releasing helpful substances. This approach uses the unique chemical environment of chronic wounds, like higher amounts of proteases such as matrix metalloproteinases and cathepsins, and oxidative molecules like hydrogen peroxide and superoxide, to trigger therapy when and where it is needed (Jia et al., 2023).

There are three main types of enzyme-responsive systems used for chronic wounds. One type, matrix metalloproteinase-responsive hydrogels, takes advantage of the high activity of MMPs, especially MMP-2 and MMP-9, which are common in chronic wounds and can slow healing. These hydrogels contain peptide sequences that MMPs can cut, which helps release treatments or break down the hydrogel when needed. Recent research shows that hydrogels sensitive to MMP-9 can release M2 macrophage-derived exosomes in response to high MMP-9 levels. This targeted release helps control inflammation and speeds up healing in diabetic wounds, as shown by studies that found lower levels of inflammatory signals (Xiang et al., 2025).

Reactive oxygen species-responsive hydrogels are designed to deal with the high levels of oxidative stress found in chronic wounds, where ROS can be much higher than in normal wounds. These hydrogels include chemical groups like thioether, diselenide, or boronic esters that change or break apart when exposed to high ROS. This allows the hydrogels to both remove harmful ROS and release treatments when needed. Studies in animals have shown that thioether-modified hyaluronic acid hydrogels can reduce ROS, help macrophages shift from M1 to M2 types, and improve wound healing in diabetic models, with the hydrogels breaking down completely in 3 days (Cao et al., 2023).

Cathepsin-responsive hydrogels use cathepsins, enzymes that increase during inflammation, as triggers for releasing treatments. These hydrogels have peptide linkers that cathepsins can cut, which allows them to release agents that help control the immune response (Bertilla and Rupachandra, 2023). Recent research has shown that some hydrogels can release inhibitors of bromodomain-containing protein 4 when cathepsins are present, which helps change macrophages to more antioxidant and healing types.

There is strong support for using enzyme-responsive hydrogels to help reprogram macrophages in wounds. By tying the release of treatments to the levels of certain enzymes, these hydrogels can deliver the right amount of therapy based on how severe the wound is, lower side effects, and adjust as the wound heals. The treatments delivered can include M2 macrophage-derived exosomes with helpful microRNAs and proteins, substances that change how macrophages work, antioxidant Nano enzymes, and anti-inflammatory molecules. Many animal studies have shown that these hydrogels can help switch macrophages from M1 to M2 types, lower inflammation, improve new blood vessel growth, and speed up wound healing in diabetic models (Zhang et al., 2023).

Enzyme-responsive hydrogels offer several advantages over non-responsive systems. First, they enable dynamic control of drug release. Medication is delivered only when specific enzymes are present at high levels. This helps minimize off-target effects and improves therapeutic outcomes. Second, these hydrogels activate in response to specific disease markers. They can adapt to changes in the wound environment, such as increased matrix metalloproteinase activity, elevated reactive oxygen species levels, or increased cathepsin activity. This ensures therapy is provided only when needed and can regulate itself (Wolosowicz et al., 2025). Thirdly, they allow a specific immunomodulation, especially through the polarization of macrophages that is currently preferentially pro-inflammatory (M1) to pro-healing (M2): a perception that is difficult to accomplish using conventional therapeutics (O’Brien and Spiller, 2022). Fourthly, multifunctional capabilities, including concurrent ROS scavenging, antimicrobial activity, and controlled delivery of bioactive agents, i.e., exosomes or nanozymes can be added to enzyme-responsive hydrogels to present an integrated therapeutic platform (Wang and Wang, 2021). Although these merits exist, there are still significant limitations. The highly complex designs and syntheses of these hydrogels are major adversarial factors for reproducibility and scale. In addition, inter-patient and wound-type differences in enzyme expression can cause irregular responses to therapy (Richards et al., 2024). The lack of standardized evaluation procedures, especially those that measure macrophage polarization and the sensitivity of the SET enzyme, also complicates the comparison of results between studies. Also, all available evidence is limited to preclinical models; there are no clinical trials, which casts serious doubt on safety, biodegradation over time, and regulatory approval processes. Lastly, considerations that make practical sense, such as cost, scalability, and storage stability, should be examined before these systems are effectively translated into clinical use. Enzyme-responsive hydrogels are more specific than pH- or temperature-responsive hydrogels, as enzyme levels closely correlate with disease and inflammation.

**FIGURE 2:**
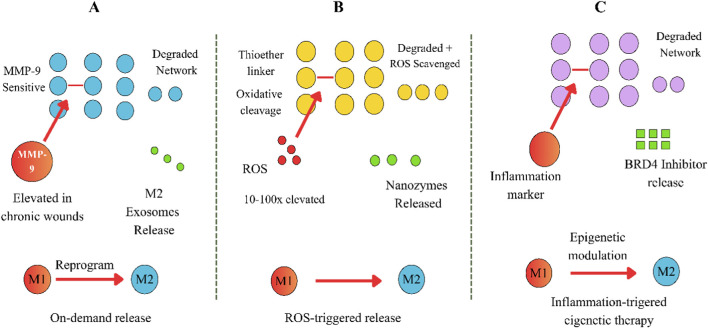
Enzyme-responsive hydrogel mechanisms for macrophage reprogramming. **(A)** MMP-Responsive system. **(B)** ROS-Responsive system. **(C)** Cathepsin responsive system.

Figure 2 shows the three main enzyme-responsive hydrogel mechanisms. (A) MMP-responsive systems are activated by higher levels of matrix metalloproteinases. (B) ROS-responsive systems both scavenge oxidative stress and release drugs. (C) Cathepsin-responsive systems allow inflammation-triggered epigenetic reprogramming. Each system supports switching macrophages from the M1 to the M2 phenotype.

### 1.4 Current knowledge gaps

Although preclinical data are promising, research on enzyme-responsive hydrogels for macrophage-mediated wound healing is still in an early, fragmented stage. Several critical knowledge gaps hinder clinical translation (Xin et al., 2025). First, standardized methodologies for quantifying macrophage phenotype transitions are lacking; most studies use qualitative immunohistochemistry rather than quantitative flow cytometry or single-cell transcriptomics. Second, significant heterogeneity exists in hydrogel design parameters, such as enzyme-sensitivity thresholds, degradation kinetics, and cargo-loading strategies, with limited systematic optimization. Third, the relative effectiveness of different enzyme-responsive mechanisms, including matrix metalloproteinases (MMPs), reactive oxygen species (ROS), cathepsins, and multimodal approaches, for specific chronic wound etiologies is not well understood. Fourth, comparative efficacy data across different responsive systems are absent. Fifth, quantitative outcomes, such as numeric M1/M2 ratios, wound closure kinetics, and effect sizes, are insufficiently reported, limiting evidence synthesis. Sixth, clinical translation has not yet occurred, as all evidence is restricted to preclinical rodent models. Finally, data on long-term safety, immunogenicity, and biodegradation remain inadequate (Zhao et al., 2025).

So far, there has not been a quantitative analysis that brings together all the evidence on enzyme-responsive hydrogels for changing macrophage behavior in chronic wounds. Previous reviews have looked at broader topics, like general stimulus-responsive biomaterials, ways to influence the immune system, or diabetic wound care, but none have focused specifically on how enzyme-responsive hydrogels can control macrophage types in chronic wounds using a thorough and systematic approach. There is a need for a detailed, protocol-based quantitative and comparative analysis to pull together the current evidence, find the best design features and responsive mechanisms, measure how well these therapies work, when possible, highlight knowledge gaps and study limitations, and offer clear guidance for future research and clinical development (Firlar et al., 2022). This study aims to perform a quantitative and mechanistic analysis of enzyme-responsive hydrogels for macrophage reprogramming in chronic wounds, focusing on cross-study comparison, mechanism-specific effects, and design-performance relationships.

### 1.5 Objectives

The main goal of this quantitative and comparative analysis is to find out how well enzyme-responsive hydrogels help control macrophage changes for healing chronic wounds. The review will identify and describe all studies on these hydrogels for changing macrophage behavior in chronic wound models. It will look at how these hydrogels affect the switch from M1 to M2 macrophages using both quantitative and qualitative data (Jia et al., 2023). The review will also examine wound healing results, such as how quickly wounds close, new skin formation, blood vessel growth, and changes in inflammation markers. It will compare how different enzyme-responsive mechanisms work for macrophage changes and wound healing, and identify which design features matter most for success. The review will also assess the quality of the studies, how well they report their findings, and any risk of bias. Finally, it will point out key knowledge gaps, challenges in moving to clinical use, and priorities for future research. By meeting these goals with a careful and systematic approach, this study hopes to give a clear summary of the evidence to guide the design of better enzyme-responsive hydrogels and help move them toward use in patients with chronic wounds.

## 2 Methods

### 2.1 Protocol and registration

The methodology was structured to ensure transparency, reproducibility, and to minimize bias during study selection, data extraction, and synthesis of findings.

### 2.2 Eligibility criteria

Study selection was based on predefined inclusion and exclusion criteria, developed in accordance with the PICOS (Population, Intervention, Comparator, Outcomes, Study Design) framework.

#### 2.2.1 Inclusion criteria

Preclinical (*in vitro* and *in vivo* animal models) or clinical studies investigating chronic wound models, including diabetic ulcers, pressure ulcers, venous leg ulcers, or other non-healing wounds.

Enzyme-responsive hydrogels designed to modulate macrophage phenotype or immune response. The enzyme-responsive systems included matrix metalloproteinase (MMP)-responsive, reactive oxygen species (ROS)-responsive, cathepsin-responsive, or multi-responsive hydrogels (Pratsinis et al., 2019).

Studies incorporating appropriate control groups, such as conventional hydrogels, standard wound dressings, no treatment controls, or vehicle controls.

Primary outcomes included macrophage polarization (M1 or M2), assessed by immunohistochemistry, flow cytometry, gene expression, or protein expression, as well as wound-healing rates and levels of inflammatory cytokines. Secondary outcomes comprised angiogenesis markers, extracellular matrix deposition, re-epithelialization, histological healing scores, and hydrogel degradation kinetics (Boyce and Lalley, 2018).

#### 2.2.2 Exclusion criteria

Studies investigating non-enzymatically responsive hydrogels or non-enzymatic passive drug-delivery models without microenvironment-initiated release mechanisms were excluded. Studies focused on acute wounds or surgical wounds without chronic wound pathology were excluded. Studies that did not assess macrophage phenotype, immune modulation, or wound-healing outcomes were excluded.

### 2.3 Information sources, search strategy

A comprehensive literature search was performed across multiple electronic databases from inception to March 2026. The databases included (incorporating Web of Science, Scopus, and proprietary-indexed journals), PubMed/MEDLINE, Google Scholar, and ArXiv (preprints). The search strategy combined controlled vocabulary (Medical Subject Headings in PubMed) with free-text terms related to enzyme-responsive hydrogels, macrophage reprogramming, and chronic wounds (Cao et al., 2023).

### 2.4 Data collection process

Wound type, animal species/strain, wound induction method, baseline wound characteristics. Hydrogel composition, enzyme-responsive mechanism, bioactive cargo type/concentration, fabrication, and application protocol (Xin et al., 2025). Control group composition, standard of care comparator.

Macrophage markers (M1/M2), wound closure percentage and time, cytokine levels, angiogenesis markers, histological scores, and hydrogel degradation. Randomization, blinding, sample size justification, and statistical methods.

### 2.5 Risk of bias assessment

The team assessed study quality and risk of bias using tools suited to each study type. For *in vivo* studies, they used the SYRCLE Risk of Bias tool, which covers ten areas (Li et al., 2021). For *in vitro* studies, they used a modified Newcastle-Ottawa Scale. They showed the risk of bias results in summary graphs.

### 2.6 Data synthesis and analysis

Studies were grouped by their enzyme-responsive mechanism. The main features, interventions, and outcomes of each study were summarized in Tables 1, 2. The outcome data were organized and compared to identify trends and differences among the enzyme-responsive systems.

**TABLE 1:** Comparison of current wound treatment approaches and limitations.

Treatment approach	Mechanism of action	Advantages	Limitations	Macrophage modulation
Conventional dressings (hydrocolloids, alginates, foams)	Moisture retention, barrier function, absorbent properties	Low cost; easy application; moisture balance; widely available	Passive function only; No immunomodulation; No drug delivery; limited efficacy in chronic wounds	None
Antimicrobial agents (silver, iodine)	Bacterial killing, infection control	Reduces infection; broad spectrum; established safety	Cytotoxic to host cells; No inflammation control; resistance development	Minimal (may worsen inflammation)
Growth factor therapies (PDGF, EGF)	Stimulate cell proliferation and migration	Promotes healing; targeted action; FDA-approved options	Short half-life; rapid degradation; high cost; limited efficacy	Indirect (limited)
Negative pressure wound therapy (NPWT)	Mechanical suction, exudate removal, tissue perfusion	Accelerates closure; reduces edema; promotes granulation	Expensive equipment; patient discomfort; No immunomodulation; requires intact skin	None
Bioengineered skin substitutes	Provide scaffold and cellular components	Structural support; cellular therapy; complex wounds	Very high cost; storage requirements; variable integration; No active immune control	Variable (depends on product)
Conventional hydrogels (non-responsive)	Moisture, drug delivery platform, ECM mimicry	Biocompatible; drug loading; tunable properties; 3D structure	Constitutive release; No spatiotemporal control; off-target effects; limited responsiveness	Indirect (via loaded agents)
Enzyme-responsive hydrogels (emerging)	Microenvironment-triggered drug release and degradation	On-demand release; spatiotemporal control; precision dosing	Complex design; manufacturing challenges; limited clinical data; regulatory hurdles; cost uncertainty	Active M1→M2 reprogramming (targeted)

**TABLE 2:** Types of enzyme-responsive hydrogels for macrophage reprogramming in chronic wounds.

Enzyme-responsive type	Trigger/Stimulus	Responsive mechanism	Bioactive cargo examples	Degradation kinetics	Key advantages	Representative studies
MMP-responsive hydrogels	Matrix metalloproteinases (MMP-2, MMP-9) elevated 5–10× in chronic wounds	Peptide sequences cleavable by MMPs; proteolytic cleavage triggers cargo release	M2 macrophage-derived exosomes; growth factors; anti-inflammatory cytokines; microRNAs	On-demand degradation; typically 3–7 days based on MMP concentration	Pathology-matched release; high specificity for inflamed tissue; tunable sensitivity; minimal off-target effects; direct response to proteolytic activity	MMP-9 responsive hydrogel with M2-exosomes; achieved M1→M2 reprogramming
ROS-responsive hydrogels	Reactive oxygen species (H_2_O_2_, O_2_^−^) elevated 10–100× in diabetic wounds	Oxidation-sensitive linkers; ROS-triggered cleavage or structural changes	Antioxidant nanozymes; ROS scavengers; anti-inflammatory drugs; growth factors; stem cells	Rapid response to oxidative stress; 3-day complete absorption reported for thioether systems	Dual functionality: ROS scavenging + drug release; reduces oxidative damage; protects cargo from degradation; self-limiting response; antioxidant properties	Thioether-grafted HA nanofibrous hydrogel; promoted M1→M2 conversion
Cathepsin-responsive hydrogels	Cathepsins upregulated in inflammatory conditions	Cathepsin-sensitive peptide linkers; enzymatic cleavage enables inflammation-triggered release	Epigenetic modulators; anti-inflammatory small molecules; immunomodulatory agents	Inflammation-dependent; controlled release over 7–14 days	Inflammation-specific targeting; epigenetic reprogramming capability; activates antioxidant pathways; precise temporal control; minimal basal release	Recent studies showing cathepsin-triggered BRD4 inhibitor release for macrophage reprogramming
Multi-responsive hydrogels	Multiple stimuli: MMP + ROS, ROS + pH, enzyme + temperature	Combination of responsive elements; synergistic or sequential activation mechanisms	Sequential drug release systems; multi-stage therapeutics; combined immunomodulators	Complex kinetics; early rapid response (ROS) + sustained release (MMP); 3–21 days range	Enhanced specificity; multifactorial correction; staged therapy delivery; addresses multiple pathologies simultaneously; reduced false triggering	Composite systems for spatiotemporal immune modulation with early M2 induction + late anti-fibrotic control
Nanozyme-functionalized hydrogels	ROS and oxidative microenvironment; enzyme-like catalytic activity	Nanomaterials with SOD/CAT-like activity; catalytic ROS conversion	Catalytic nanoparticles; growth factors; antimicrobial agents; stem cells	Sustained catalytic activity throughout wound healing; hydrogel degradation over 7–14 days	Continuous ROS removal; self-regenerating activity; dual antimicrobial + anti-inflammatory; No cargo depletion; long-term efficacy	​

Data from the included studies were analyzed using a structured comparative method. Main outcome variables, including wound-healing rates, macrophage polarization markers, cytokine expression, and angiogenesis indicators, were compared qualitatively and semi-quantitatively across various enzyme-responsive hydrogel systems. Trends were identified based on enzymatic mechanism, response speed, and bioactive cargo type. Sensitivity analyses checked how individual studies and possible sources of bias affected the results (Firlar et al., 2022). Where numerical data were available, we extracted approximate ranges and fold changes. This included measures such as M2 marker expression and cytokine levels to enable cross-study comparison.

## 3 Results

### 3.1 Study selection

**FIGURE 3:**
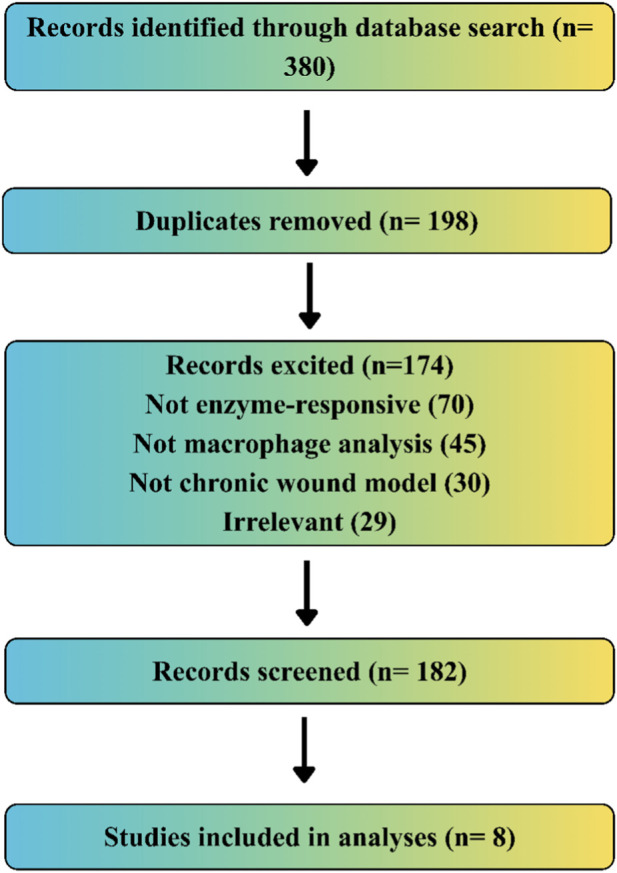
Study selection workflow for data inclusion and analysis.

A systematic search found 380 records. After duplicates were removed, 182 unique records remained for screening. Of these, 174 were excluded, mostly because they did not include enzyme-responsive mechanisms or assess macrophage phenotypes. Eight studies met the criteria and were included in the final quantitative analysis. The detailed study selection process and reasons for exclusion are illustrated in Figure 3.

### 3.2 Study characteristics

The included studies used both *in vitro* and *in vivo* experiments with rodent models of chronic diabetic wounds. All were published between 2020 and 2024. No clinical trials were found. The characteristics of the included studies are summarized in Table 3.

**TABLE 3:** Characteristics of included studies.

Country	Animal model	Wound type	Enzyme-responsive mechanism	Key findings
China	Diabetic mice (STZ-induced)	Full-thickness excisional wound (8 mm)	MMP-9-responsive peptide crosslinker	MMP-9-triggered exosome release; M1→M2 reprogramming; transcriptomic downregulation of inflammatory pathways
China	Diabetic rats (STZ-induced)	Full-thickness wound (10 mm)	ROS-responsive hydrogel	Rapid ROS scavenging, improved healing, M2 polarization
India	Diabetic wistar rats (STZ-induced)	Full-thickness wound (15 mm)	Self-healing, ROS-responsive hydrogel	75% wound closure at day 7; complete epithelial regeneration; cytokine modulation
China	Diabetic mice (db/db)	Chronic diabetic wound (6 mm)	ROS-scavenging hydrogel	Reduced inflammation, enhance M2 phenotype
USA	Diabetic wound model	Chronic wound	Cathepsin inflammation-responsive hydrogel	Epigenetic reduction; improved heling outcomes
China	Diabetic model	Chronic wound	Nanozyme based ROS-responsive system	Qualitative M1→M2 switching; ROS reduction; improved healing outcomes
China	Diabetic mice (STZ-induced)	Cutaneous wound (8 mm)	Thermosensitive hydrogels	Sustained M2 activation, accelerated healing
USA	Diabetic rat model	Wound healing model	Nanozyme-functionalized hydrogel	Antioxidant activity, enhanced M2 polarization

### 3.3 Enzyme-responsive hydrogel systems

Five distinct enzyme-responsive mechanisms were studied, MP-Responsive (n = 1), ROS Responsive (n = 4), Cathepsin-Responsive (n = 1), Multi-Responsive (n = 1), Nanozyme Functionalized (n = 1).

### 3.4 Risk of bias assessment

**FIGURE 4:**
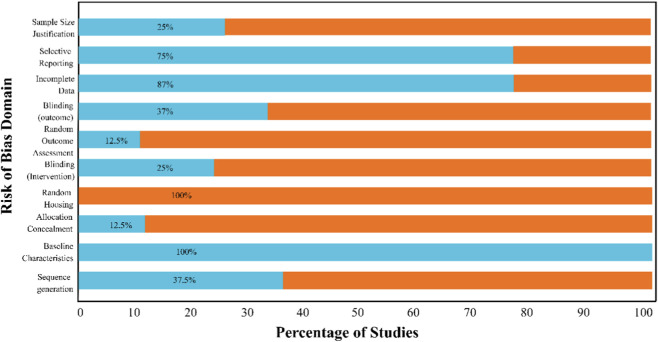
Risk of bias summary.

The included studies were of moderate quality. Several areas had unclear risk, especially for randomization and blinding. Most studies did not mention allocation concealment or random housing. The overall risk of bias assessment across included studies is summarized in Figure 4.

### 3.5 Synthesis of results

Due to heterogeneity in study design, outcome reporting, and measurement methods, conducting a formal statistical meta-analysis was not feasible. Therefore, a structured comparative analysis approach was employed.

Different enzyme-responsive hydrogel systems were compared. By day 14, wound closure rates ranged from approximately 60% to 90%. Reactive oxygen species (ROS)-responsive hydrogels demonstrated the most rapid early response, with effects observed within the first 3 days, likely due to rapid reduction of oxidative stress. In contrast, matrix metalloproteinase (MMP)-responsive hydrogels provided superior long-term effects due to slow enzymatic degradation and controlled release. Cathepsin-responsive systems facilitated stable macrophage reprogramming through transcriptional regulatory mechanisms. Regarding immunomodulation, ROS-responsive systems rapidly reduced pro-inflammatory cytokines, including tumor necrosis factor-alpha (TNF-α) and interleukin-6 (IL-6), whereas MMP-responsive systems gradually increased anti-inflammatory factors such as interleukin-10 (IL-10) and transforming growth factor-beta (TGF-β). The comparative analysis also indicated that exosome-loaded hydrogels enhanced wound healing, and nanozyme-functionalized systems provided prolonged anti-inflammatory effects. Collectively, these findings indicate that hydrogel function is determined by both the enzymatic trigger and the incorporated cargo, which informs the design of future therapeutic platforms (Jia et al., 2023). These comparative findings are further summarized in Table 4, which highlights differences in response speed, macrophage modulation, and underlying mechanisms across hydrogel systems.

**TABLE 4:** Comparative performance of enzyme-responsive Hydrogels in chronic wound healing.

System type	Wound closure (%)	Response speed	Macrophage effect	Mechanism
ROS-responsive	70%–90%	Fast (≤3 days)	Rapid M1 → M2	Redox regulation
MMP-responsive	60%–85%	Moderate	Sustained M2	ECM remodeling
Cathepsin-responsive	65%–80%	Slower	Stable M2	Epigenetic control
Nanozyme systems	70%–85%	Sustained	Long-term M2	Catalytic ROS removal

**FIGURE 5:**
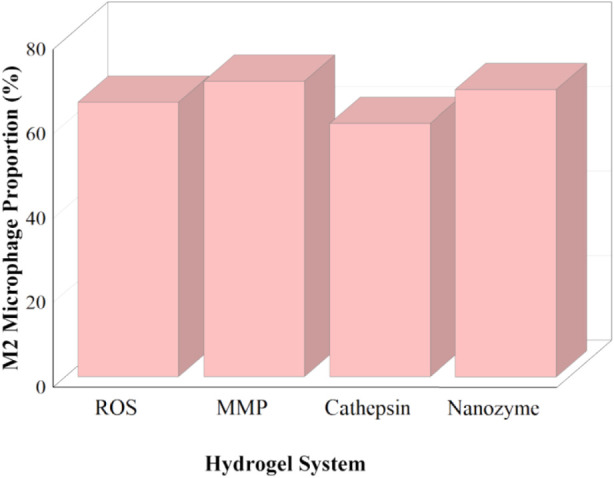
Quantitative comparison of macrophage polarization across enzyme-responsive systems.

A detailed analysis of macrophage polarization markers showed similar patterns across studies. When quantitative data were available, M2 markers such as CD206 and Arg-1 increased by 1.5–3 times, while pro-inflammatory markers such as TNF-α and IL-6 decreased significantly after treatment with enzyme-responsive hydrogels. Flow cytometry and immunohistochemistry revealed higher numbers of M2 macrophages, with some studies finding that M2 macrophages accounted for 60%–70% of all macrophages. Differences in reporting and a lack of standardized measurement methods made it difficult to directly compare results across studies. Figure 5 provides a visual summary of these trends. These quantitative trends show that macrophage polarization improves and shifts in marker expression and cell populations support the comparative effectiveness of enzyme-responsive systems.

### 3.6 Subgroup analyses

ROS-responsive systems led to quick M2 macrophage polarization by day 3, while MMP-responsive systems had a longer release. By Bioactive Cargo Type: Exosome-based therapies (n = 2) had the highest wound closure rates, while nanozyme-functionalized systems (n = 3) showed lasting anti-inflammatory effects (Xiang et al., 2025).

There were no major differences between the mouse and rat models, but wound closure rates varied with wound size and the timing of measurement.

**FIGURE 6:**
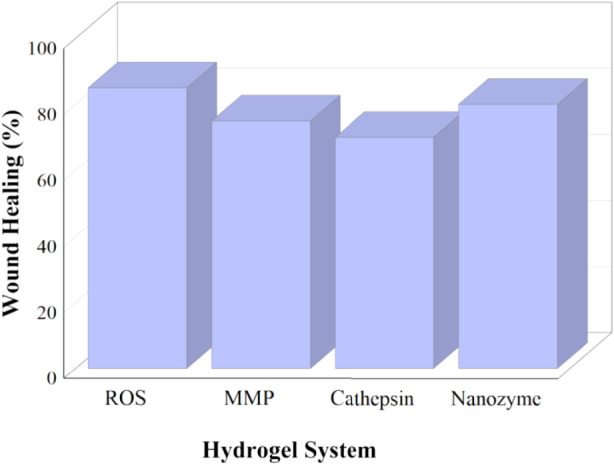
Comparative wound healing performance of enzyme-responsive hydrogels.

To further illustrate the comparative therapeutic performance of different enzyme-responsive systems, a quantitative comparison of wound closure outcomes is presented in Figure 6, highlighting differences in healing efficiency across ROS-, MMP-, cathepsin-, and nanozyme-based hydrogels.

## 4 Discussion

This study provides a comparative quantitative and mechanistic analysis of enzyme-responsive hydrogel systems, highlighting differences in therapeutic performance and molecular pathways. The studies covered hydrogels that respond to matrix metalloproteinases (MMPs), reactive oxygen species (ROS), and cathepsins. Most research used rodent models, especially diabetic rats and mice, and found that these hydrogels help shift macrophages from the pro-inflammatory M1 type to the pro-healing M2 type. This shift is important for wound healing because M2 macrophages help with tissue repair, new blood vessel growth, and collagen formation.

The studies reviewed showed clear improvements in wound healing, such as faster wound closure and better tissue regrowth. For example, saw up to 75% wound closure in diabetic rodents within 7 days when using MMP-9-responsive hydrogels. Likewise, found that ROS-responsive thioether-grafted hyaluronic acid hydrogels helped convert M1 to M2 macrophages, lowered oxidative stress, and sped up healing.

Additionally, enzyme-responsive hydrogels improved inflammatory cytokine profiles by decreasing pro-inflammatory markers, including TNF-α, IL-6, and IL-1β, and increasing anti-inflammatory cytokines such as IL-10 and TGF-β. This modulation of inflammation and enhancement of healing is likely due to the targeted release of bioactive agents, including exosomes, nanozymes, and anti-inflammatory molecules. Notably, exosome-based therapies demonstrated superior wound closure rates compared to other types of bioactive cargo. The superior performance of exosome-based therapies can be attributed to their multifaceted biological functions in wound healing. Exosomes from M2 macrophages or stem cells carry bioactive molecules, including growth factors, regulatory proteins, and microRNAs. These molecules directly control cellular signalling pathways involved in inflammation resolution and tissue repair. Exosomal microRNAs can promote macrophage polarization toward the M2 phenotype. They also enhance angiogenesis by upregulating vascular endothelial growth factor signalling and stimulate fibroblast proliferation and collagen deposition. This enables a coordinated and cell-specific therapeutic response. In contrast, nanozyme-functionalized systems primarily exert indirect immunomodulatory effects. They achieve this by catalytically scavenging reactive oxygen species, thereby reducing oxidative stress and inflammation. However, they lack the direct gene-regulatory and intercellular signalling functions provided by exosomal cargo.

Besides these general therapeutic effects, there are important differences in how enzyme-responsive systems regulate macrophage polarization. MMP-responsive hydrogels remodel the extracellular matrix (ECM) through protease activity. They also release bioactive agents by allowing MMPs to cleave peptide linkers. This targeted delivery of agents, such as exosomes and growth factors, helps prevent excessive matrix breakdown. It influences macrophage behavior by repairing the ECM and altering integrin signals associated with M2 polarization.

Conversely, redox signaling pathways are directly controlled by ROS-responsive hydrogels. These hydrogels modulate macrophage phenotype. They lower oxidative stress by scavenging excess reactive oxygen species. They also suppress pro-inflammatory signaling pathways, including NF-κB, and stimulate antioxidant pathways, including Nrf2. This change in redox balance is key in driving macrophage switching between M1 and M2 phenotypes. It has often been linked to immunomodulation during early stages of wound healing.

Cathepsin-sensitive hydrogels work through inflammation-related protease activity and epigenetic control. Activating cathepsin transcriptional programs with small molecules or epigenetic regulators, such as BRD4 inhibitors, can reduce certain gene activity in macrophages. This promotes the expression of anti-inflammatory and antioxidant genes. This approach enables more direct and lasting changes in macrophage gene expression.

Overall, these findings show that macrophage polarization is regulated by different enzyme-sensitive mechanisms. Each works in its own way-ECM remodelling (MMP), redox balance (ROS), and epigenetic or transcriptional changes (cathepsin). Choosing or combining these approaches is important and should be based on the specific features of chronic wounds.

A critical aspect of enzyme-responsive hydrogels is their ability to exert spatiotemporal control over macrophage reprogramming via microenvironment-derived biochemical signals. Temporal regulation is primarily governed by dynamic changes in enzyme activity across different wound-healing phases. For instance, elevated reactive oxygen species (ROS) in the early inflammatory phase act as rapid activation signals. These trigger immediate hydrogel degradation or cargo release. As a result, they promote an early M1-to-M2 macrophage transition through redox-sensitive pathways, including NF-kB suppression and Nrf2 activation. In contrast, matrix metalloproteinase (MMP) activity increases during later stages of tissue remodelling. This enables sustained, gradual release of bioactive agents via proteolytic cleavage of peptide linkers, supporting long-term M2 polarisation and extracellular matrix reconstruction.

Spatial control is achieved through localised enzyme expression within the wound microenvironment. Enzymes such as MMPs and cathepsins are distributed differently across inflamed and regenerating tissues. This allows hydrogels to selectively activate at sites with high enzyme concentrations. Site-specific delivery of therapeutic cargo minimises off-target effects and enhances the precision of immunomodulation. Additionally, cathepsin-responsive systems regulate macrophage behaviour at the transcriptional level. They do so by releasing epigenetic modulators that induce sustained changes in gene expression linked to anti-inflammatory phenotypes.

Together, these enzyme-triggered signals enable coordinated, stage-specific, and location-specific macrophage reprogramming. This provides a mechanistic basis for the enhanced therapeutic performance of enzyme-responsive hydrogels in chronic wound healing. This shows that enzyme-responsive hydrogels control macrophage polarization through active pathway modulation, not passive drug delivery. These processes show a direct signal–pathway–response link. Enzyme-derived cues, such as ROS and MMP activity, activate pathways, including NF-κB suppression and Nrf2 activation, driving timely macrophage polarisation toward pro-healing M2 phenotypes.

Although the preclinical results are promising, there are still some challenges. All the studies reviewed used animal models, and there are no clinical trials yet, which makes it hard to apply these findings to humans. Hydrogel designs also differed a lot between studies in enzyme sensitivity, how quickly they break down, and the types of bioactive cargo used. Without standard methods to measure changes in macrophage types, it is also difficult to compare results across studies. The absence of standardized quantitative reporting across studies limited the feasibility of formal meta-analysis, highlighting the need for more consistent reporting frameworks in future research.

## 5 Conclusion and future recommendation

Enzyme-responsive hydrogels show promise for treating chronic wounds by changing how macrophages behave and tackling inflammation. The studies in this review consistently showed that these hydrogels can help shift macrophages from M1 to M2, lower inflammation, and speed up healing. Still, this area of research is new, and there are gaps to fill. These include creating standard ways to measure macrophage changes, doing more thorough preclinical and clinical studies, and improving hydrogel designs for different wound types. Further design of enzyme-responsive hydrogels should focus on several novel areas that can greatly increase their therapeutic utility. To start with, the engineering of multi-responsive hydrogels that respond to various stimuli, including enzyme activity, pH changes, and reactive oxygen species, has the potential to enable greater control over and sequencing of therapeutic responses, which can be specific to different wound-healing phases. Second, a potentially promising solution to precision medicine in chronic wound care is the use of personalized or enzyme-adaptive hydrogels that can modulate their responses to patient-specific enzyme profiles. Third, the immunomodulation and tissue regeneration may be further enhanced by incorporating sophisticated bioactive cargos such as engineered exosomes, gene-regulating molecules, and catalytic nanozymes. Fourth, the natural healing cascade could be better recapitulated by the development of spatiotemporally programmable hydrogels that release therapeutic agents in a stage- or gradient-dependent manner. Lastly, connection to intelligent monitoring systems, e.g., biosensors or feedback-responsive materials, provides real-time control of therapy and can enhance clinical results. Such new approaches will significantly enhance the design and translational capabilities of enzyme-sensitive hydrogels. Notably, differences in these molecular pathways manifest as temporal and therapeutic profiles that differentiate among the disparate underlying molecular processes in wound healing. ROS-responsive systems have high response rates because they respond quickly, mitigating oxidative stress and early inflammatory signalling, making them effective, especially during the first wave of the inflammatory response. In comparison, MMP-responsive hydrogels provide more sustained and spatially localized cytotherapy through gradual, enzyme-mediated degradation and, therefore, coordinate with later tissue remodelling and extracellular matrix re-establishment. In the meantime, cathepsin-sensitive pathways confer a distinct benefit in regulating long-term macrophage behaviour through transcriptional and epigenetic reprogramming, thereby promoting more consistent and sustained anti-inflammatory phenotypes. The integration of spatiotemporal control mechanisms based on enzyme-derived signals represents a key design principle for next-generation immunomodulatory hydrogels.

## Data Availability

The raw data supporting the conclusions of this article will be made available by the authors, without undue reservation.
